# Molecularly Designed and Nanoconfined Polymer Electronic Materials for Skin-like Electronics

**DOI:** 10.1021/acscentsci.4c01541

**Published:** 2024-11-18

**Authors:** Yu-Qing Zheng, Zhenan Bao

**Affiliations:** † Department of Chemical Engineering, Stanford University, Stanford, California 94305, United States; ‡ National Key Laboratory of Advanced Micro and Nano Manufacture Technology; Beijing Advanced Innovation Center for Integrated Circuits, School of Integrated Circuits, Peking University, Beijing 100871, China

## Abstract

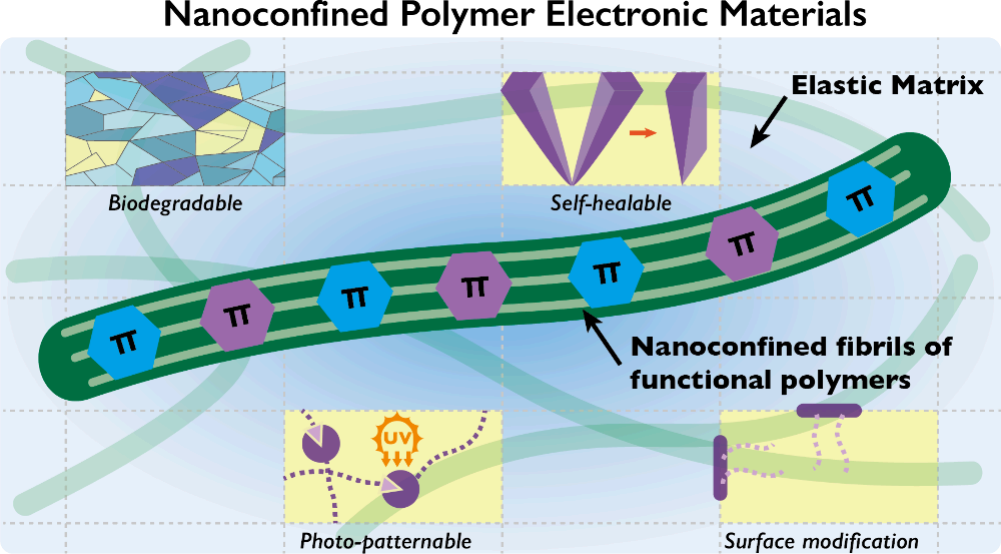

Stretchable electronics have seen substantial development in skin-like mechanical properties and functionality thanks to the advancements made in intrinsically stretchable polymer electronic materials. Nanoscale phase separation of polymer materials within an elastic matrix to form one-dimensional nanostructures, namely nanoconfinement, effectively reduces conformational disorders that have long impeded charge transport properties of conjugated polymers. Nanoconfinement results in enhanced charge transport and the addition of skin-like properties. In this Outlook, we highlight the current understanding of structure–property relationships for intrinsically stretchable electronic materials with a focus on the nanoconfinement strategy as a promising approach to incorporate skin-like properties and other functionalities without compromising charge transport. We outline emerging directions and challenges for intrinsically stretchable electronic materials with the aim of constructing skin-like electronic systems.

## Abstract 2

Nanoconfinement reduces conformational disorder in functional polymers, enhancing charge transport and enabling multiple skin-like properties through a tailored elastic matrix.

## Introduction

Skin is the largest organ of the body, which protects our body while allowing us to sense the surroundings. It has a unique ability to withstand tensile strain and compression while retaining normal functions. Inspired by the sensing, signal processing, and materials properties, of human skin, we have been developing electronic skins (E-skins) to mimic skin functions and properties, such as stretchability, self-healing ability, and biodegradability. This is now an active subject of research. E-skin may enable revolutionary developments in precision health,^1,2^ prosthetics,^3−5^ humanoid robots,^6,7^ human-machine interface, artificial intelligence and virtual/augmented realities. To achieve skin-like stretchability with rigid electronics, geometric engineering, such as incorporating serpentine or Kirigami interconnects, rigid islands, or wrinkled structures, has been used to protect rigid components from mechanical damage.^8−10^ However, this strategy may sacrifice device density and interfacial failure between mechanically mismatched rigid and soft materials.

To address this challenge, molecularly engineered polymer materials can be used to imbue electronic devices with stretchability and other skin-like properties, such as self-healing ability, biodegradability, etc., eventually leading to high-density integrated, large-area and mechanically robust E-skin systems.^11,12^ Therefore, materials are key to the development of skin-like intrinsically stretchable electronics.^13,14^ However, despite the inherent flexibility of polymer materials, the molecular design and morphology requirements for high stretchability and good electrical charge transport properties contradict each other.^15,16^ Improving stretchability requires more flexible polymer backbones and more disordered polymer thin film morphology, which usually result in poor electrical charge transport properties and poor device performance. Moreover, imparting multiple skin-like properties or beyond, such as micropattern ability, environmental stability, etc., into a single material is challenging as each of these functions requires distinct molecular design principles and morphology tuning.

As the understanding of structure–property relationships in polymer materials continues to be advanced, several promising molecular design strategies for high-performance intrinsically stretchable polymer materials have been reported, all of which are based on the general goal of balancing the ratios between ordered and disordered domains to improve mechanical stretchability while only mildly decreasing charge transport capability.^17^ Alternatively, a nanoconfinement strategy, involving formation of one-dimensional (1D) conjugated polymer nanostructures by blending a polymer electronic material with a matrix polymer network with skin-like properties, has stood out in terms of outstanding performance and multifunctionalities.^18^ In this Outlook, we discuss the energy dissipation mechanisms for polymer materials under strain, which forms the foundation for the discussion of molecular design strategies and a nanoconfinement approach for high-performance intrinsically stretchable polymer electronic materials. Then, we discuss the general applicability of the nanoconfinement strategy to incorporate various skin-like properties as well as desirable material features for device fabrication and robust device operation.

## Energy Dissipation Mechanisms in Conjugated Polymers under Strain

In the quest to unlock electronic stretchable materials and devices on a macroscopic scale, a comprehension of the microscopic behaviors of conjugated polymers is imperative. Stretchability, more specifically, elasticity, describes the ability of polymer networks to experience a mechanical deformation without fracture and then return to their original shape when the strain is removed. Elastomers, such as silicone rubber (polydimethylsiloxane, PDMS) and styrene–butadiene rubber (SBR), are stretchable and return to their original shape below certain strain levels.^19,20^ When stretched, the chain segments of polymers in a rubbery state (i.e., amorphous state) undergo conformational changes to straighten and then align the polymer chains along the strain direction.^21,22^ This mechanism of energy dissipation gives macroscopic mechanical deformation without irreversible aggregation and crystalline domain breakage or covalent bond breakage. Therefore, the stretchability of polymers is closely related to their molecular design, including polymer backbone design, side chain structures and substitution positions as well as degree of polymerization (i.e., molecular weight) and polydispersity, which impact their microscopic conformations, microstructures and crystallinity.^23^ Generally, a high molecular weight and/or a lower persistence length (i.e., more flexible polymer chain) tend to have a higher fraction of amorphous domains, while large crystalline domains and high crystallinity may result in brittle films and irreversible deformation.^24^ Relative stretchability (*rS*), defined as the ratio of change in dichroic ratio to the change in relative degree of crystallinity (rDOC) under strain, has been found to provide good prediction of the ability of conjugated polymer thin films to maintain charge transport properties under strain.^24^ The advantage of this parameter compared to other parameters, such as Young’s modulus and tensile strength, is that it captures the morphological features and changes of conjugated polymer thin films used directly for corresponding devices. These features are usually dependent on the processing conditions.

Different from conventional rubber polymers, conductive and semiconducting polymers are based on rigid aromatic conjugated backbone structures.^25^ Charge transport in these polymers is facilitated by the delocalization of charge carriers along the conjugated backbone (i.e., intrachain transport) as well as through hopping between π–π stacked chains (i.e., interchain transport).^26^ Thus, to achieve high charge carrier mobilities, stiff and planar conjugated polymer backbone structures are desirable for crystalline packing to realize good intrachain and interchain transport. Such a molecular design requirement is against the desirable molecular features and morphology for high stretchability and low modulus.^27^ Therefore, equipping conjugated polymers with both good charge transport and stretchability is challenging.^28,29^ Not surprisingly, a survey of 51 conjugated polymers showed those with fused aromatic rings had higher modulus, while bulky side chains lowered crystallinity and thus led to lower modulus.^30^

**Figure 1:**
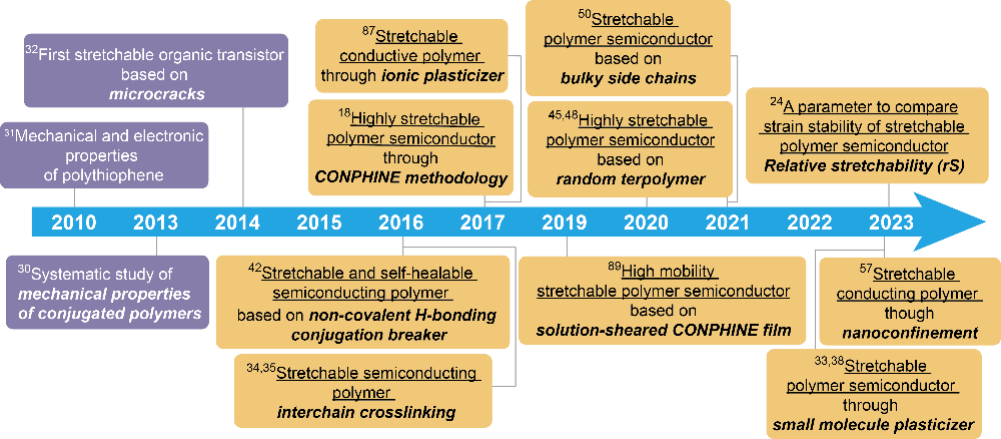
Key time points along the development path of stretchable electronic materials. Purple text boxes denote significant advances in studies prior to the development of chemically engineered stretchable electronic materials. Yellow text boxes highlight key breakthroughs in intrinsically stretchable electronic materials.

**Figure 2:**
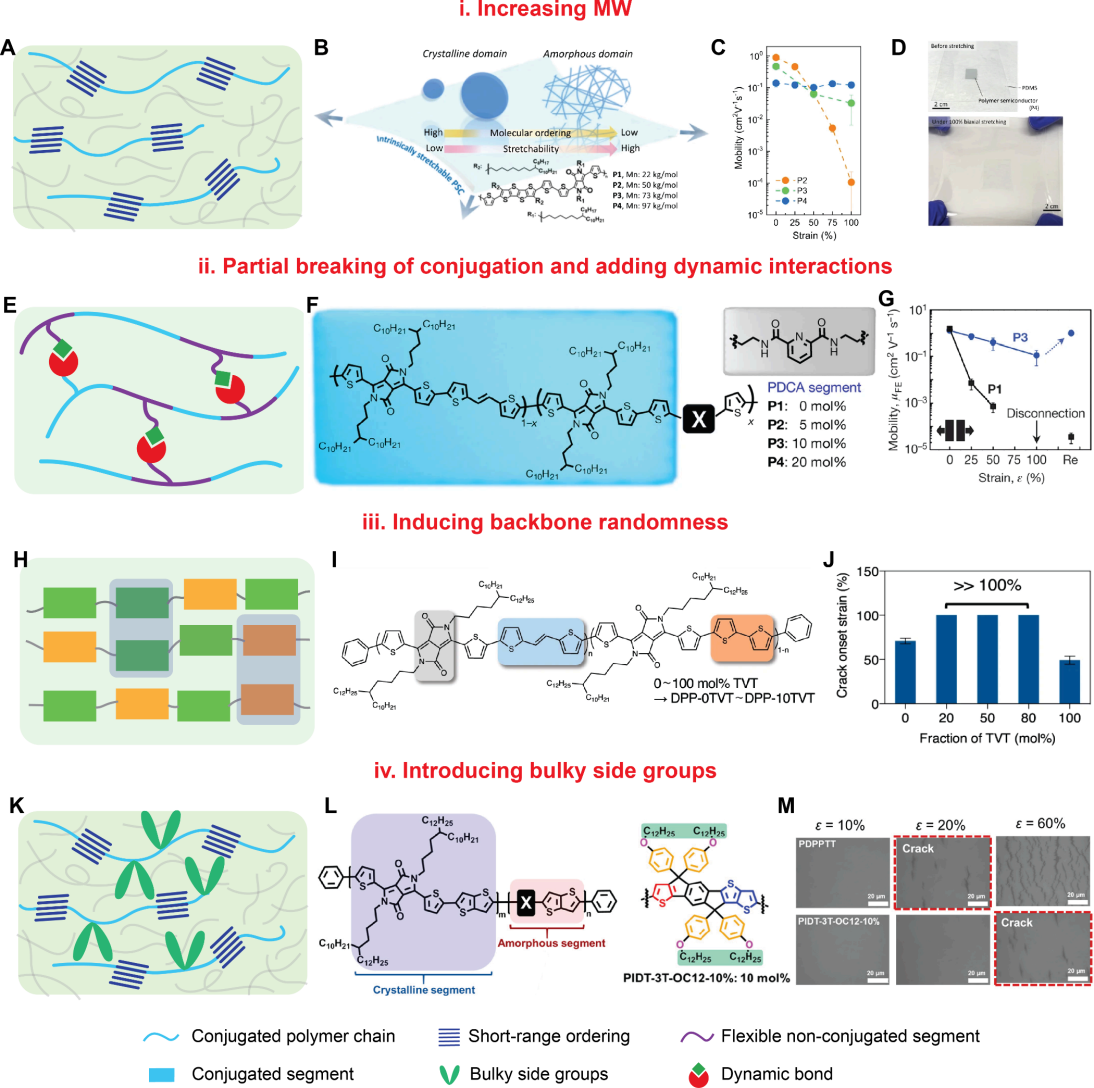
Schematic examples of molecular design strategies to realize both high carrier mobilities and high stretchability. (a) Schematic illustration of the microstructure of conjugated polymer tie chains connecting small aggregates. This type of morphology may be realized with high MW polymers. (b) Schematic of the impact of microstructure on conjugated polymer thin film stretchability. Reproduced from ref (^24^).^24^ Available under a CC-BY (Creative Commons Attribution 4.0 International license) license. Copyright 2023 H.-C. Wu et al. (c) Average charge carrier mobilities of polymers from (b) with different MWs under various biaxial strains. Reproduced from ref (^24^).^24^ Available under a CC-BY (Creative Commons Attribution 4.0 International license) license. Copyright 2023 H.-C. Wu et al. (d) Photograph of a high MW polymer film under 100% biaxial strain. Reproduced from ref (^24^).^24^ Available under a CC-BY (Creative Commons Attribution 4.0 International license) license. Copyright 2023 H.-C. Wu et al. (e) Schematic illustration of adding dynamic interactions. (f) Chemical structures of conjugated polymers with dynamic bonding units. Reprinted with permission from ref (^42^).^42^ Copyright 2016 Springer Nature. (g) Charge carrier mobilities of polymers with or without dynamic bonding units under various strains. Reprinted with permission from ref (^42^).^42^ Copyright 2016 Springer Nature. (h) Schematic illustration of microstructure of terpolymer. (i) Chemical structures of terpolymers. Reproduced from ref (^48^).^48^ Available under a CC-BY (Creative Commons Attribution 4.0 International license) license. Copyright 2021 J. Mun et al. (j) Crack onset strain of the terpolymers. Reproduced from ref (^48^).^48^ Available under a CC-BY (Creative Commons Attribution 4.0 International license) license. Copyright 2021 J. Mun et al. (k) Schematic illustration of the microstructure of conjugated polymers with bulky side groups. (l) Chemical structures of conjugated polymers with bulky side chains. Reprinted with permission from ref (^50^).^50^ Copyright 2021 American Chemical Society. (m) Optical microscopy images of conjugated polymers with and without bulky side groups under strain. Reprinted with permission from ref (^50^).^50^ Copyright 2021 American Chemical Society.

Despite the challenges, substantial progress has been made on the molecular design of high-performance intrinsically stretchable conjugated polymers, leveraging the collective progress in understanding of synthetic rubber materials, dynamic chemistry, polymer semiconductors, and polymer mechanics (Figure 1).^31−38^ First, more recent findings on charge transport in high mobility polymers suggested that short-range order together with polymer tie chains bridging aggregates are effective in realizing good charge transport, making high crystallinity not an essential requirement.^39−41^ Based on this consideration, high molecular weight conjugated polymers are expected to have both good mechanical stretchability and good charge transport (Figure 2a). Indeed, a high molecular weight diketopyrrolopyrrole (DPP)-based polymer semiconductor, P2TBDPP2TBFT4 (P4, chemical structure shown in Figure 2b) showed a high *rS* of 11.5 and high tolerance to biaxial strain up to 100%. In contrast, another more crystalline low molecular weight DPP polymer semiconductor, PDPPTVT, had an *rS* of only 0.38 with crack formation even at low strains (5%–10%).^42^ Remarkably, P2TBDPP2TBFT4 was found to have a morphology similar to well-known thermoplastic elastomers, that is with small-sized aggregates within a largely amorphous polymer network, in which morphology readily recovered after strain was removed.^24^

## Molecular Design Strategies toward High-Performance Intrinsically Stretchable Conjugated Polymers

Molecular design strategies have endowed conjugated polymers with stretchability and high mobility by finely tuning the morphology and microstructures of the conjugated polymer thin films. These strategies include increasing molecular weight (MW) as discussed above,^43−45^ partial breaking of conjugation,^42,46,47^ increasing backbone randomness,^45,48^ and introducing bulky side chains^49,50^ (Figure 2).

### (i). Partial Breaking of Conjugation and Adding Dynamic Interactions

Earlier studies showed that DPP-based copolymers with a small amount of nonconjugated backbone spacers maintained a high hole mobility of over 1 cm^2^ V^–1^ s^–1^.^51,52^ We showed that adding a small fraction of conjugation breaker (5–10 mol %) can effectively reduce the elastic modulus of the polymer by almost ten times.^46^ Furthermore, we used a hydrogen-bonding conjugation breaker to form a noncovalent cross-linked polymer network for additional energy dissipation mechanisms under strain (Figure 2e and f).^42^ The flexible alkyl segment alone could only extend the crack formation onset strain from ∼5%–10% to 30% due to its primary energy dissipation mechanism of crystallite breaking (*rS* = 0.45) (Figure 2g). In contrast, with 10 mol % of nonconjugated hydrogen-bond-forming monomers, high mobilities (>1 cm^2^ V^–1^ s^–1^), high stretchability (crack onset strain >100%), and highly efficient healing ability of electrical performance were realized in a single semiconducting polymer (*rS* increased to 2.65).

### (ii). Increase Backbone Randomness

Introducing multiple comonomers into DPP to give so-called terpolymers is a convenient way to induce backbone randomness.^45,48,50^ Both high mobility of over 1 cm^2^ V^–1^ s^–1^ and greatly improved stretchability with crack onset strain >100% were realized. These properties are attributed to the presence of short-range aggregations with reduced overall crystallinity and average crystalline domain sizes (Figure 2h and i). On the contrary, regular DPP D–A copolymers are more crystalline and can experience severe fracturing at less than 25% strain (Figure 2j). Introducing backbone randomness is readily applicable to various donors and acceptors with different combinations, such as [Donor]-[Acceptor 1]-[Acceptor 2] or [Acceptor]-[Donor 1]-[Donor 2].

### (iii). Introducing Bulky Side Groups

Aside from tuning the polymer backbone structures, side chains can also strongly affect the microstructure of conjugated polymer thin films by bringing about additional intermolecular interactions.^25^ To find a balance between mobility and stretchability, we needed to finely tune polymer thin film crystallinity. Introducing a fraction of conjugated rigid fused-rings with bulky side groups into high-mobility DPP polymers reduces the strong aggregation tendency between DPP polymer backbones (Figure 2k and l).^50^ This resulted in thin films with a much decreased rDOC and improved stretchability with up to 60% crack onset strain (Figure 2m).

In summary, the above molecular design strategies have pushed the mobilities of stretchable conjugated polymers to a level similar to, or in some cases surpassing, that of amorphous silicon. The mobilities can be well maintained under repeated external strain. A limitation of these strategies is that they require an iterative design-synthesis-characterization processes to identify suitable polymers. On the other hand, there are continued discoveries of new higher-performing high mobility polymers reported in the literature. It would therefore be desirable to have a strategy that can be applied to a broad range of polymers to impart stretchability without compromising or even boosting the charge carrier mobility. Therefore, an engineering strategy that can be applied to existing high-performance polymers off the shelf will accelerate the development of stretchable electronics.

## Nanoconfinement Effect for High-Performance Intrinsically Stretchable Polymers

Blends of conducting nanomaterials in a polymer matrix have been widely used for stretchable conductors.^53^ For example, carbon nanotubes, silver nanowires, and silver flakes are common conductive fillers. For stretchable semiconductors, poly(3-hexylthiophene) (P3HT) nanofibrils pregrown in a cooled P3HT and poly(styrene)-poly(ethylene butylene)-poly(styrene) (SEBS) solution phase mixture was found to phase-segregate to the solution–air interface.^54^ The resulting thin films had a much decreased mobility compared to neat P3HT, mostly due to the interruption of charge transport by the insulating components. Nevertheless, with the addition of the SEBS elastomer, the films retained 30% of their mobility under 50% external strain. The cycling reliability of such films was limited with a significant drop of on/off current after being stretched for 10 cycles due to breakage of the nanofibrils under stress.

The examples presented above illustrate the widely used blending strategy, where one material with one function is dispersed into another with a second function to gain both functions in the blend material. While this approach has been effective in achieving some degree of stretchability and semiconducting properties, the electrical performances of the material usually substantially degrade. A recent discovery of the nanoconfinement effect of the functional electronic polymer within the matrix successfully addressed the above issue to result in both enhanced electronic and mechanical properties.

**Figure 3:**
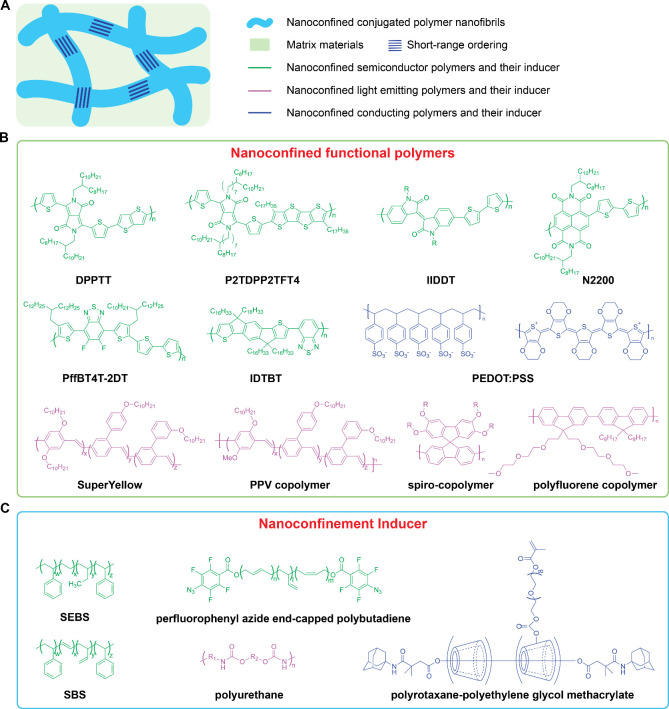
Examples of nanoconfined polymer systems. (a) Schematic illustration of the nanoconfinement strategy. Nanoconfined fibrils are formed directly and are interconnected inside a deformable matrix. Nanoconfinement is important for enhanced charge transport through reducing conformational disorder while an interconnected network is important to both achieve and maintain good charge transport in the strained thin film. (b) Representative functional polymers that produce nanoconfined elastic semiconductors. (c) Representative matrix materials used to induce nanoconfinement in conjugated polymers. The colors of the chemical structures are chosen to match the corresponding conjugated polymers used in nanoconfinement.

Nanoconfinement structures are nanoconfined fibrils of conjugated polymers within an elastomer matrix. They may be obtained from the phase separation of conjugated and insulating polymer blends. It was found that the addition of an appropriate elastic matrix material may induce nanoconfined conjugated polymer structures to give stretchable semiconducting films with outstanding electrical performance (conjugated-polymer/elastomer phase separation-induced elasticity (CONPHINE)) (Figure 3a). While more detailed mechanistic studies are still ongoing, it was found that the nanoconfined structures were induced in solution state by the addition of the insulating polymer.^18^ The nanoconfined structures in solution have diameters of a few nanometers, much smaller than the persistence length of most conjugated polymers. It was observed that the nanoconfined structures in solution further aggregated into large fibril structures with the conjugated polymer chains aligned along the long axis of the fibrils, while the conjugated polymer solution without addition of an elastomer exhibited random nucleation and growth to give highly disordered thin films. To further control the nanoscale phase separation in these films, both thermodynamic and kinetic factors must be considered. Miscibility between two components controls the thermodynamic process of nanoconfinement, while processing parameters, such as solvent, coating speed, thermal annealing temperature, and deposition techniques, control the kinetic process of polymer aggregation and crystallization in nanoconfinement systems. The latter is especially important for multicomponent polymer blending systems and for achieving interconnected fibril networks to facilitate good charge transport throughout the film. The effects of solvent boiling point were systematically studied on the phase separation and correlation with macroscopic device performance.^55^ It was shown that the rapid evaporation of low boiling-point solvents was beneficial for nanoscale liquid–liquid phase separation, which can enhance the aggregation of conjugated chains via the nanoconfinement effect, while the slow evaporation of high boiling-point solvents resulted in an undesirable increase in conjugated polymer domain sizes. Moreover, compared to spin-cast composites, using solution shearing with a comb-shaped blade, introduced macroscale alignment of the nanoconfined conjugated polymer fibrils in the elastomer composites yielded a threefold increase in carrier mobility, as well as an almost unchanged transfer curve under strain up to 100%.^56^

Nanoconfinement led to enhanced polymer chain dynamics and suppressed crystallization while maintaining short-range aggregation and reduced conformational disorder. Unlike typical blends, in which charge transport becomes worse, the CONPHINE films showed a higher charge carrier mobility compared to the neat conjugated polymer due to reduction of conformational disorder.^18^ The CONPHINE strategy additionally greatly enhanced stretchability up to 100% strain while enabling increased mobility over 1 cm^2^ V^–1^ s^–1.^ Thin film transistor devices using CONPHINE as the active layer could endure repeated stretching, twisting, and even poking by sharp objects. Under 100% strain, the on-current of the CONPHINE film was three orders of magnitude higher than that of the corresponding neat conjugated polymer. Since field-effect transistor charge transport only occurs within a few nanometers of the semiconductor/dielectric interface,^57^ the vertical phase separation formed in SEBS/DPP-based polymer blend film was beneficial for good ohmic contacts and charge transport. A series of studies about the CONPHINE methodology based on combinations of various classes of conjugated polymers,^58−72^ elastomers,^58−61,72−76^ and polymers with different MWs,^77−80^ proved that a nanoconfined polymer semiconductor in a thermoplastic elastomer was a general way to reduce conformational disorder and incorporate skin-inspired properties while boosting electrical performance, such as charge carrier mobility,^81^ conductivity,^59^ thermoelectric properties,^82^ photoluminescence, photodetection,^83^ memory performance,^70^ sensing performance,^63^ etc (Figure 3b and c).

To take advantage of the nanoconfinement effect for other optoelectronic materials with various chemical structures, the interactions with the matrix polymers should be carefully tuned. For example, poly(3,4-ethylenedioxythiophene) polystyrenesulfonate (PEDOT:PSS) aqueous solution is commercially available and widely used to prepare conducting polymer thin films with high conductivity greater than 1000 S/cm (Figure 3b).^84^ Although the PEDOT:PSS thin film is flexible, it is also brittle and forms cracks with only a 5% elongation.^85^ However, the hydrophobic elastomers used for inducing nanoconfinement in polymer semiconductors are not suitable for PEDOT:PSS, which is a PSS-stabilized PEDOT dispersion in water. On the other hand, ionic liquids, water-soluble polyethylene glycol (PEG) and its derivatives are successful examples to endow PEDOT:PSS with intrinsic stretchability by the nanoconfinement strategy.^59,86−89^ In a PEG-based supramolecular cross-linked elastic matrix, the crystallization of PEG was suppressed by encapsulating it within the bulky cyclodextrins around the PEG chains. Additional short PEG side chains further promoted nanoscale phase separation while providing sites for photo-cross-linkable groups to give an elastic matrix with embedded nanoconfined PEDOT and therefore improved stretchability (Figure 3c). Such nanoconfined PEDOT:PSS films can reach up to 2700 S/cm conductivity as well as an impressive stretchability with only a 5 times resistance increase under 150% strain.^59^

A uniform distribution along both the vertical and horizontal directions is required for continuous vertical charge transport in light-emitting diodes and photodetector devices. Polyurethane (PU) has been found to be a suitable matrix polymer for a series of light-emitting conjugated polymers (Figure 3c).^58^ In addition to added stretchability, the photoluminescence efficiency and LED current density could be enhanced by the nanoconfinement strategy due to improved charge transport and trap dilution effects.^58^ Using the stretchable nanoconfined PEDOT:PSS as transparent electrodes, the stretchable all-polymer light-emitting diode achieved brightness up to about 7,450 cd m^–2^ and stretchability of around 100% strain.

## Nanoconfinement Enabled Multifunctional Intrinsically Stretchable Polymers

**Figure 4:**
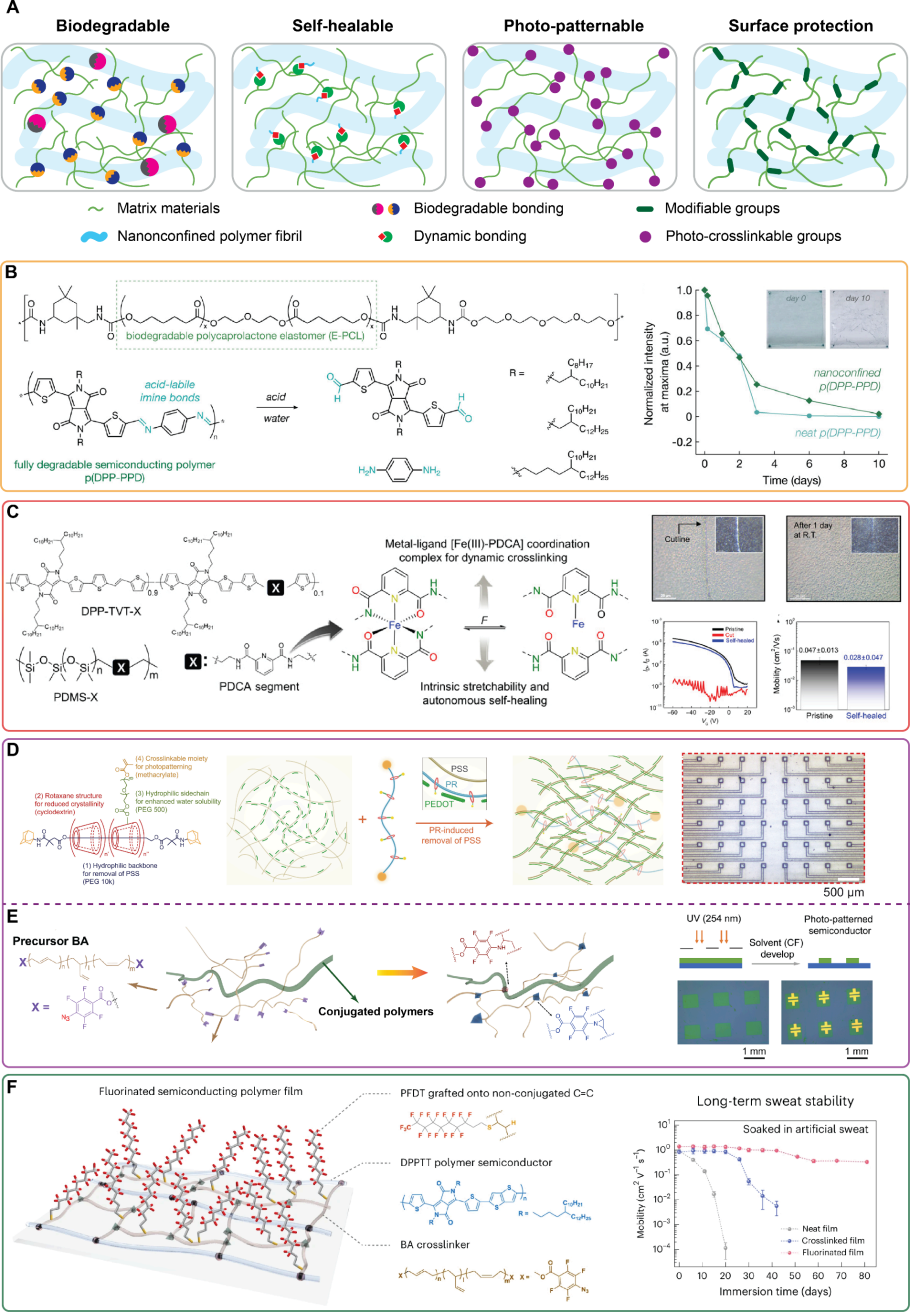
Examples of multifunctional stretchable polymer films based on the nanoconfinement strategy. (a) Schematics of the nanoconfinement strategy to realize biodegradability, self-healing ability, photopatterning ability, and surface functionalization/encapsulation in skin-like polymer electronic materials. (b) Chemical structures of the biodegradable elastomer and the degradable semiconducting polymer, and degradability of the nanoconfined film exemplified by the decrease of UV–vis absorption. Reproduced from ref (^90^).^90^ Available under a CC-BY license. Copyright 2019 H. Tran et al. (c) Chemical structures of dynamic bonding moieties introduced into the semiconducting polymer and matrix polymer and the self-healing ability of these nanoconfinement systems. Reproduced from ref (^91^).^91^ Available under a CC BY-NC (Creative Commons Attribution NonCommercial License 4.0) license. Copyright 2019 American Association for the Advancement of Science. (d) Chemical structures of photo-cross-linkable matrix molecules for PEDOT:PSS and optical microscope image of photo-patterned nanoconfined PEDOT:PSS. Reprinted with permission from ref (^59^).^59^ Copyright 2022 American Association for the Advancement of Science. (e) Mechanism of photopatterning ability of semiconducting polymer by selective cross-linking of perfluorophenyl azide end-capped polybutadiene matrix. Reprinted with permission from ref (^92^).^92^ Available under a Creative Commons CC BY license. Copyright 2021 Y. Zheng et al. (f) Schematic and long-term sweat stability of environmentally robust nanoconfined semiconducting film achieved by covalently bonded fluorinated molecules onto the nonconjugated C=C bond of the rubber matrix. Reprinted with permission from ref (^93^).^93^ Copyright 2023 Springer Nature.

The nanoconfinement effect provides a general approach toward multifunctional stretchable polymer electronic materials by incorporating the desirable functions into the matrix polymer. This enabled significant broadening of the scope of functions achievable with high-performance conjugated polymers without having to go through extensive optimization of molecular design to identify suitable polymers with all of the required functions. Frequently, it is not possible to simultaneously achieve good charge transport and additional functionalities. The nanoconfinement approach has enabled additional incorporation of biodegradability,^90^ self-healing ability,^74,76,91^ photopatterning ability,^59,92,94,95^ environmental stability,^93^ and bioadhesion^96^ into stretchable high-performance electronic polymers (Figure 4a). Notably, through the rational design of the matrix polymers, minimal negative impacts on electronic performance were observed with nanoconfinement systems. For example, to demonstrate stretchable and fully degradable semiconductors, we incorporated urethane units into the matrix polymer and reversible imine bonds into the semiconducting polymer for degradability (Figure 4b).^90^ The nanoconfined film could be degraded under acidic aqueous conditions within 10 days.

A similar strategy has also been effective in creating stretchable and self-healable semiconductors. Inspired by the autonomous repairing ability of human skin, self-healing polymer materials that can reconnect and regain their electronic properties after multiple instances of damage are crucial to prolonging the lifetime of the E-skin. The chemical basis of self-healing is dynamic noncovalent interactions such as hydrogen bonding, metal–ligand coordination, and ionic interactions.^95−98^ These bonds dissociate under external force and reassociate when polymer chains are in contact again spontaneously by diffusion or passively by pressing together the two fractured surfaces.^99,100^ A series of self-healable and stretchable dielectric and semiconducting polymers based on dynamic hydrogen-bonding or metal–ligand interactions have been successfully developed.^42,101−105^ Blending of conventional or self-healable semiconducting polymers with a suitable self-healable elastomer can result in a nanoconfined conjugated polymer network in a supramolecular dynamic cross-linked network (Figure 4c).^74,76,91^ Upon screening the blending ratio, the nanoconfined semiconducting system could maintain high stretchability and also enable autonomous self-healing at room temperature. DPP-based conjugated polymer/amide-functionalized polyisobutylene (PIB) blends showed stable mobilities up to 150% strain and could be recovered 100% within 15 min after scratching.^74^ In contrast, the neat semiconducting polymer component could only be healed with the help of solvent post-treatment,^42^ due to the lowered glass transition temperature by the matrix polymer.

In addition to skin-like properties, other requirements need to be considered for these materials. Since the matrix component is the major component in the nanoconfined system, it could be used to fulfill these requirements. With the increasing promise of E-skin for high-resolution mapping of signals, miniaturization of sensors and signal processing components has become an important topic. Micropatterning of intrinsically stretchable electronic polymers is critical. By incorporating photoactivatable chemical groups (such as methacrylate,^59,95^ azide,^92^ diazirine,^95^ thiol^94^) to matrix materials in nanoconfined films, upon photoactivation, a chemically cross-linked elastic matrix could be formed in situ in the polymer blend film. During solvent development, functional polymers could be well preserved within the cross-linked matrix (Figure 4d and e). The direct photopatterning ability of intrinsically stretchable nanoconfinement systems endowed by a photo-cross-linkable matrix is appealing because (i) the patterning process is simplified without the need for a protective layer or photoresist removal or etching, (ii) the patterning process does not degrade electrical properties if properly engineered, and (iii) it could be applied to various functional polymer materials by designing the appropriate matrix. High patterning resolution down to 2 μm was successfully demonstrated in PEDOT:PSS blends (Figure 4d). High elasticity as well as photopatternability were realized to give a stretchable polymer semiconductor film via a covalently embedded in situ rubber matrix (iRUM) (Figure 4e).^92^ Furthermore, taking advantage of the nonsaturated bonds in the matrix polymer, fluorination protection could be applied to substantially improve the environmental stability of the devices and maintain good electrical and mechanical properties (Figure 4f).^93^ The resulting semiconductor film had low water permeability and stable operation in harsh environments, including water, artificial sweat, and simulated sunlight irradiation in air. The charge carrier mobilities could be well maintained up to 1 cm^2^ V^–1^ s^–1^ in artificial sweat for 42 days. This encapsulation strategy is an effective approach for maintaining intrinsically stretchable device performance under various conditions. It may find applications in wearable and implantable devices.

## Future Outlook

Chemical insights and molecular design are critical in enabling the new generation of skin-like polymer electronic materials. Benefiting from the synergistic progress in fabrication and characterization, the electrical performance of intrinsically stretchable polymer electronic materials are now on par with those reported for flexible electronics, while intrinsically stretchable polymers also offer additional desirable properties for direct interfacing with biological systems such as low modulus, high stretchability, self-healing ability, and biodegradability. The mobilities of stretchable polymer semiconductors have reached a level similar to (or even surpassed that of) amorphous silicon and can be well maintained under repeated external strain. The conductivities of stretchable conducting polymer thin films similar to those of indium tin oxide, an inorganic transparent electrode material, have been realized. Understanding molecular design rules and intricate interactions in multicomponent polymer blends to induce nanoconfinement effects in conjugated polymers has been essential in enabling fundamental advancements. In particular, the nanoconfinement discovery resulted in much reduced conformational disorder in conjugated polymers that is critical for realizing high charge carrier mobility, high electrical conductivity, and dilution of trap density for light-emission. The nanoconfinement effect is a general method that allows adding skin-like properties, photopatternability, and even an effective encapsulation strategy while maintaining or boosting electrical performance. Furthermore, the nanoconfinement structures can be controlled through solution-state aggregates and in situ nanoscale phase separation during film formation, which makes it possible to scale with large-area fabrication.

Going forward, there remain important fundamental and engineering challenges to address. Since skin-inspired electronics have a broad range of potential applications, the above understanding will need to be developed in the context of particular targeted potential applications. But broadly speaking, the development of nanoconfined materials with even better mechanical and electronic properties will be grounded in the continued understanding of its formation process and structure–property relationships through novel molecular design of both functional materials and matrix materials and advanced microstructure characterization technologies.

(1) The formation of nanoconfinement structures in blend systems is governed by both thermodynamic and kinetic processes. It is therefore important to perform advanced characterizations to gain detailed information on nanoconfined structures in solution and their assembly process, including nucleation and growth processes of the functional component within the matrix. While most studies to-date have focused on elucidating the final microstructures, the processes leading to the formation of these nanoconfined structures remain underexplored. To address this gap, in-depth in situ characterization techniques will be essential. Techniques such as in situ UV–Vis spectroscopy, in situ grazing incidence wide-angle X-ray scattering (GIWAXS) can continuously capture the process of assembly, while others, such as cryogenic electron microscopy (cryo-EM), can freeze the intermediate stages for detailed analysis. By gaining a full picture of the formation process, we can potentially achieve precise control over the size, length, orientation, degree of order and connectivity of the nanoconfined fibers by adjusting the chemical structures of each component or processing parameters.
(2) A range of functional materials has been successfully combined with matrix polymers and showed the nanoconfinement effect, providing valuable insights into selecting appropriate matrices for specific functional materials. However, even small variations in chemical structures, molecular weight, polydispersity index, or changes in the mixing ratio can significantly impact the nanoconfined structures and their connectivity. As a result, extensive experimental conditions need to be tested to optimize the morphology and charge transport. This is where autonomous lab and machine learning (ML) may help to accelerate the development to identify the ideal material combinations for achieving optimal nanoconfinement morphology.
(3) Aside from electrical properties, mechanical properties including long cycling stability under mechanical deformation, multidirection stretchability, high mechanical toughness, room-temperature autonomous self-healing ability, and programmable biodegradability, are subjects requiring further molecular level understanding for robust operation of skin-like electronics in practical applications. For example, understanding the energy dissipation mechanism at the molecular-level can provide guidance on the specific nanoconfined microstructure network morphology needed to achieve targeted stretchability. Alongside, accurate quantitative characterizations of mechanical behaviors, such as stress–strain curves, is crucial for understanding the multidimensional mechanical performance of nanoconfined thin films. This goes beyond simply using crack onset strain as a measure of stretchability. These detailed insights are essential for optimizing the performance and durability of next-generation electronic skins under real-world conditions.

## References

[ref1] Gao W.; Ota H.; Kiriya D.; Takei K.; Javey A. Flexible Electronics toward Wearable Sensing. Acc. Chem. Res. 2019, 52 (3), 523–533. 10.1021/acs.accounts.8b00500. 30767497

[ref2] Sugiyama M.; Uemura T.; Kondo M.; Akiyama M.; Namba N.; Yoshimoto S.; Noda Y.; Araki T.; Sekitani T. An Ultraflexible Organic Differential Amplifier for Recording Electrocardiograms. Nat. Electron. 2019, 2 (8), 351–360. 10.1038/s41928-019-0283-5.

[ref3] Shin M.; Oh J. Y.; Byun K.-E.; Lee Y.-J.; Kim B.; Baik H.-K.; Park J.-J.; Jeong U. Pursuing Prosthetic Electronic Skin. Adv. Mater. 2015, 27 (7), 1255–1261. 10.1002/adma.201404602. 25581228

[ref4] Amoli V.; Kim J. S.; Jee E.; Chung Y. S.; Kim S. Y.; Koo J.; Choi H.; Kim Y.; Kim D. H. A Bioinspired Hydrogen Bond-Triggered Ultrasensitive Ionic Mechanoreceptor Skin. Nat. Commun. 2019, 10 (1), 4019 10.1038/s41467-019-11973-5. 31488820 PMC6728325

[ref5] Yu Y.; Nassar J.; Xu C.; Min J.; Yang Y.; Dai A.; Doshi R.; Huang A.; Song Y.; Gehlhar R.; Ames A. D.; Gao W. Biofuel-Powered Soft Electronic Skin with Multiplexed and Wireless Sensing for Human-Machine Interfaces. Sci. Robot. 2020, 5 (41), eaaz7946 10.1126/scirobotics.aaz7946. 32607455 PMC7326328

[ref6] Gao G.; Chang C.-M.; Gerez L.; Liarokapis M. A Pneumatically Driven, Disposable, soft robotic gripper equipped with retractable, telescopic fingers. IEEE Trans. Med. Robot. Bionics 2021, 3, 573–582. 10.1109/TMRB.2021.3097143.

[ref7] Guo Y.; Zhong M.; Fang Z.; Wan P.; Yu G. A Wearable Transient Pressure Sensor Made with MXene Nanosheets for Sensitive Broad-Range Human-Machine Interfacing. Nano Lett. 2019, 19 (2), 1143–1150. 10.1021/acs.nanolett.8b04514. 30657695

[ref8] Savagatrup S.; Printz A. D.; O’Connor T. F.; Zaretski A. V.; Lipomi D. J. Molecularly Stretchable Electronics. Chem. Mater. 2014, 26 (10), 3028–3041. 10.1021/cm501021v.

[ref9] Kim D.-H.; Lu N.; Ma R.; Kim Y.-S.; Kim R.-H.; Wang S.; Wu J.; Won S. M.; Tao H.; Islam A.; Yu K. J.; Kim T.; Chowdhury R.; Ying M.; Xu L.; Li M.; Chung H.-J.; Keum H.; McCormick M.; Liu P.; Zhang Y.-W.; Omenetto F. G.; Huang Y.; Coleman T.; Rogers J. A. Epidermal Electronics. Science 2011, 333 (6044), 838–843. 10.1126/science.1206157. 21836009

[ref10] Kaltenbrunner M.; Sekitani T.; Reeder J.; Yokota T.; Kuribara K.; Tokuhara T.; Drack M.; Schwödiauer R.; Graz I.; Bauer-Gogonea S.; Bauer S.; Someya T. An Ultra-Lightweight Design for Imperceptible Plastic Electronics. Nature 2013, 499 (7459), 458–463. 10.1038/nature12314. 23887430

[ref11] Trung T. Q.; Lee N. Recent Progress on Stretchable Electronic Devices with Intrinsically Stretchable Components. Adv. Mater. 2017, 29 (3), 1603167 10.1002/adma.201603167. 27862355

[ref12] Wang S.; Oh J. Y.; Xu J.; Tran H.; Bao Z. Skin-Inspired Electronics: An Emerging Paradigm. Acc. Chem. Res. 2018, 51 (5), 1033–1045. 10.1021/acs.accounts.8b00015. 29693379

[ref13] Wagner S.; Bauer S. Materials for Stretchable Electronics. MRS Bull. 2012, 37 (3), 207–213. 10.1557/mrs.2012.37.

[ref14] Kim D. C.; Shim H. J.; Lee W.; Koo J. H.; Kim D. Material-Based Approaches for the Fabrication of Stretchable Electronics. Adv. Mater. 2020, 32 (15), 1902743 10.1002/adma.201902743. 31408223

[ref15] Tien H.-C.; Huang Y.-W.; Chiu Y.-C.; Cheng Y.-H.; Chueh C.-C.; Lee W.-Y. Intrinsically Stretchable Polymer Semiconductors: Molecular Design, Processing and Device Applications. J. Mater. Chem. C 2021, 9 (8), 2660–2684. 10.1039/D0TC06059C.

[ref16] Zheng Y.; Zhang S.; Tok J. B.-H.; Bao Z. Molecular Design of Stretchable Polymer Semiconductors: Current Progress and Future Directions. J. Am. Chem. Soc. 2022, 144 (11), 4699–4715. 10.1021/jacs.2c00072. 35262336

[ref17] Xue X.; Li C.; Shangguan Z.; Gao C.; Chenchai K.; Liao J.; Zhang X.; Zhang G.; Zhang D. Intrinsically Stretchable and Healable Polymer Semiconductors. Adv. Sci. 2024, 11 (8), 2305800 10.1002/advs.202305800. PMC10885676 38115748

[ref18] Xu J.; Wang S.; Wang G.-J. N.; Zhu C.; Luo S.; Jin L.; Gu X.; Chen S.; Feig V. R.; To J. W. F.; Rondeau-Gagné S.; Park J.; Schroeder B. C.; Lu C.; Oh J. Y.; Wang Y.; Kim Y.-H.; Yan H.; Sinclair R.; Zhou D.; Xue G.; Murmann B.; Linder C.; Cai W.; Tok J. B.-H.; Chung J. W.; Bao Z. Highly Stretchable Polymer Semiconductor Films through the Nanoconfinement Effect. Science 2017, 355 (6320), 59–64. 10.1126/science.aah4496. 28059762

[ref19] Reincke W.; Grellman B. Structure-property correlations of SSBR/BR blends. In Deformation and Fracture Behaviour of Polymer Materials, 1st ed.; Springer Nature: Cham, 2017; pp 461–466.

[ref20] Liu M.; Sun J.; Sun Y.; Bock C.; Chen Q. Thickness-Dependent Mechanical Properties of Polydimethylsiloxane Membranes. J. Micromech. Microeng. 2009, 19, 035028 10.1088/0960-1317/19/3/035028.

[ref21] Erman B.; Mark J. E. The molecular basis of rubberlike elasticity. In The Science and Technology of Rubber, 4^th^ ed; Elsevier Inc.: Netherlands, 2013; pp 167–192.

[ref22] Erman B.; Mark J. E. Rubberlike elasticity. In Polymer Science: A Comprehensive Reference, 1st ed.; Elsevier B.V.: Netherlands, 2012; pp 181–186.

[ref23] Rubinstein M.; Colby R. H. Polymer microstructure. In Polymer Physics, 1st ed.; Oxford University Press: Oxford, 2003; pp 2–5.

[ref24] Wu H.-C.; Nikzad S.; Zhu C.; Yan H.; Li Y.; Niu W.; Matthews J. R.; Xu J.; Matsuhisa N.; Arunachala P. K.; Rastak R.; Linder C.; Zheng Y.-Q.; Toney M. F.; He M.; Bao Z. Highly Stretchable Polymer Semiconductor Thin Films with Multi-Modal Energy Dissipation and High Relative Stretchability. Nat. Commun. 2023, 14 (1), 8382 10.1038/s41467-023-44099-w. 38104194 PMC10725446

[ref25] Kim M.; Ryu S. U.; Park S. A.; Choi K.; Kim T.; Chung D.; Park T. Donor-Acceptor-Conjugated Polymer for High-Performance Organic Field-Effect Transistors: A Progress Report. Adv. Funct. Mater. 2020, 30 (20), 1904545 10.1002/adfm.201904545.

[ref26] Bronstein H.; Nielsen C. B.; Schroeder B. C.; McCulloch I. The Role of Chemical Design in the Performance of Organic Semiconductors. Nat. Rev. Chem. 2020, 4, 66–77. 10.1038/s41570-019-0152-9. 37128048

[ref27] Klauk H. Organic semiconductor materials for transistors. In Organic Electronics II; WILEY-VCH Verlag GmbH & Co. KGaA: Weinheim, 2012; pp 1–23.

[ref28] Chen A. X.; Kleinschmidt A. T.; Choudhary K.; Lipomi D. J. Beyond Stretchability: Strength, Toughness, and Elastic Range in Semiconducting Polymers. Chem. Mater. 2020, 32 (18), 7582–7601. 10.1021/acs.chemmater.0c03019.

[ref29] Fan X.; Nie W.; Tsai H.; Wang N.; Huang H.; Cheng Y.; Wen R.; Ma L.; Yan F.; Xia Y. PEDOT:PSS for Flexible and Stretchable Electronics: Modifications, Strategies, and Applications. Adv. Sci. 2019, 6 (19), 1900813 10.1002/advs.201900813. PMC6774040 31592415

[ref30] Roth B.; Savagatrup S.; de Los Santos N. V.; Hagemann O.; Carlé J. E.; Helgesen M.; Livi F.; Bundgaard E.; Søndergaard R. R.; Krebs F. C.; Lipomi D. J. Mechanical Properties of a Library of Low-Band-Gap Polymers. Chem. Mater. 2016, 28 (7), 2363–2373. 10.1021/acs.chemmater.6b00525.

[ref31] O’Connor B.; Chan E. P.; Chan C.; Conrad B. R.; Richter L. J.; Kline R. J.; Heeney M.; McCulloch I.; Soles C. L.; DeLongchamp D. M. Correlations between Mechanical and Electrical Properties of Polythiophenes. ACS Nano 2010, 4 (12), 7538–7544. 10.1021/nn1018768. 21080648

[ref32] Chortos A.; Lim J.; To J. W. F; Vosgueritchian M.; Dusseault T. J.; Kim T.-H.; Hwang S.; Bao Z. Adv. Mater. 2014, 26, 4253–4259. 10.1002/adma.201305462. 24740928

[ref33] Xu J.; Wu H.; Mun J.; Ning R.; Wang W.; Wang G. N.; Nikzad S.; Yan H.; Gu X.; Luo S.; Zhou D.; Tok J. B. -H.; Bao Z. Tuning Conjugated Polymer Chain Packing for Stretchable Semiconductors. Adv. Mater. 2022, 34 (22), 2104747 10.1002/adma.202104747. 34558121

[ref34] Wang G.-J. N.; Zheng Y.; Zhang S.; Kang J.; Wu H.-C.; Gasperini A.; Zhang H.; Gu X.; Bao Z. Tuning the Cross-Linker Crystallinity of a Stretchable Polymer Semiconductor. Chem. Mater. 2019, 31 (17), 6465–6475. 10.1021/acs.chemmater.8b04314.

[ref35] Wang G. N.; Gasperini A.; Bao Z. Stretchable Polymer Semiconductors for Plastic Electronics. Adv. Electron. Mater. 2018, 4 (2), 1700429 10.1002/aelm.201700429.

[ref36] Matsuhisa N.; Chen X.; Bao Z.; Someya T. Materials and Structural Designs of Stretchable Conductors. Chem. Soc. Rev. 2019, 48 (11), 2946–2966. 10.1039/C8CS00814K. 31073551

[ref37] Wang G.-J. N.; Shaw L.; Xu J.; Kurosawa T.; Schroeder B. C.; Oh J. Y.; Benight S. J.; Bao Z. Inducing Elasticity through Oligo-Siloxane Crosslinks for Intrinsically Stretchable Semiconducting Polymers. Adv. Funct. Mater. 2016, 26 (40), 7254–7262. 10.1002/adfm.201602603.

[ref38] Mun J.; Kang J.; Zheng Y.; Luo S.; Wu H.; Matsuhisa N.; Xu J.; Wang G. N.; Yun Y.; Xue G.; Tok J. B. -H.; Bao Z. Conjugated Carbon Cyclic Nanorings as Additives for Intrinsically Stretchable Semiconducting Polymers. Adv. Mater. 2019, 31 (42), 1903912 10.1002/adma.201903912. 31489716

[ref39] Noriega R.; Rivnay J.; Vandewal K.; Koch F. P. V.; Stingelin N.; Smith P.; Toney M. F.; Salleo A. A General Relationship between Disorder, Aggregation and Charge Transport in Conjugated Polymers. Nat. Mater. 2013, 12 (11), 1038–1044. 10.1038/nmat3722. 23913173

[ref40] Zhang X.; Bronstein H.; Kronemeijer A. J.; Smith J.; Kim Y.; Kline R. J.; Richter L. J.; Anthopoulos T. D.; Sirringhaus H.; Song K.; Heeney M.; Zhang W.; McCulloch I.; DeLongchamp D. M. Molecular Origin of High Field-Effect Mobility in an Indacenodithiophene-Benzothiadiazole Copolymer. Nat. Commun. 2013, 4 (1), 2238 10.1038/ncomms3238. 23900027

[ref41] Venkateshvaran D.; Nikolka M.; Sadhanala A.; Lemaur V.; Zelazny M.; Kepa M.; Hurhangee M.; Kronemeijer A. J.; Pecunia V.; Nasrallah I.; Romanov I.; Broch K.; McCulloch I.; Emin D.; Olivier Y.; Cornil J.; Beljonne D.; Sirringhaus H. Approaching Disorder-Free Transport in High-Mobility Conjugated Polymers. Nature 2014, 515 (7527), 384–388. 10.1038/nature13854. 25383522

[ref42] Oh J. Y.; Rondeau-Gagné S.; Chiu Y.-C.; Chortos A.; Lissel F.; Wang G.-J. N.; Schroeder B. C.; Kurosawa T.; Lopez J.; Katsumata T.; Xu J.; Zhu C.; Gu X.; Bae W.-G.; Kim Y.; Jin L.; Chung J. W.; Tok J. B.-H.; Bao Z. Intrinsically Stretchable and Healable Semiconducting Polymer for Organic Transistors. Nature 2016, 539 (7629), 411–415. 10.1038/nature20102. 27853213

[ref43] Rodriquez D.; Kim J.-H.; Root S. E.; Fei Z.; Boufflet P.; Heeney M.; Kim T.-S.; Lipomi D. J. Comparison of Methods for Determining the Mechanical Properties of Semiconducting Polymer Films for Stretchable Electronics. ACS Appl. Mater. Interfaces 2017, 9 (10), 8855–8862. 10.1021/acsami.6b16115. 28220705

[ref44] Pei D.; Wang Z.; Peng Z.; Zhang J.; Deng Y.; Han Y.; Ye L.; Geng Y. Impact of Molecular Weight on the Mechanical and Electrical Properties of a High-Mobility Diketopyrrolopyrrole-Based Conjugated Polymer. Macromolecules 2020, 53 (11), 4490–4500. 10.1021/acs.macromol.0c00209.

[ref45] Lin Y.-C.; Huang Y.-W.; Hung C.-C.; Chiang Y.-C.; Chen C.-K.; Hsu L.-C.; Chueh C.-C.; Chen W.-C. Backbone Engineering of Diketopyrrolopyrrole-Based Conjugated Polymers through Random Terpolymerization for Improved Mobility-Stretchability Property. ACS Appl. Mater. Interfaces 2020, 12 (45), 50648–50659. 10.1021/acsami.0c14592. 33138353

[ref46] Mun J.; Wang G. N.; Oh J. Y.; Katsumata T.; Lee F. L.; Kang J.; Wu H.; Lissel F.; Rondeau-Gagné S.; Tok J. B. -H.; Bao Z. Effect of Nonconjugated Spacers on Mechanical Properties of Semiconducting Polymers for Stretchable Transistors. Adv. Funct. Mater. 2018, 28 (43), 1804222 10.1002/adfm.201804222.

[ref47] Savagatrup S.; Zhao X.; Chan E.; Mei J.; Lipomi D. J. Effect of Broken Conjugation on the Stretchability of Semiconducting Polymers. Macromol. Rapid Commun. 2016, 37 (19), 1623–1628. 10.1002/marc.201600377. 27529823

[ref48] Mun J.; Ochiai Y.; Wang W.; Zheng Y.; Zheng Y.-Q.; Wu H.-C.; Matsuhisa N.; Higashihara T.; Tok J. B.-H.; Yun Y.; Bao Z. A Design Strategy for High Mobility Stretchable Polymer Semiconductors. Nat. Commun. 2021, 12 (1), 3572 10.1038/s41467-021-23798-2. 34117254 PMC8196107

[ref49] Chiang Y.-C.; Wu H.-C.; Wen H.-F.; Hung C.-C.; Hong C.-W.; Kuo C.-C.; Higashihara T.; Chen W.-C. Tailoring Carbosilane Side Chains toward Intrinsically Stretchable Semiconducting Polymers. Macromolecules 2019, 52 (11), 4396–4404. 10.1021/acs.macromol.9b00589.

[ref50] Liu D.; Mun J.; Chen G.; Schuster N. J.; Wang W.; Zheng Y.; Nikzad S.; Lai J.-C.; Wu Y.; Zhong D.; Lin Y.; Lei Y.; Chen Y.; Gam S.; Chung J. W.; Yun Y.; Tok J. B.-H.; Bao Z. A Design Strategy for Intrinsically Stretchable High-Performance Polymer Semiconductors: Incorporating Conjugated Rigid Fused-Rings with Bulky Side Groups. J. Am. Chem. Soc. 2021, 143 (30), 11679–11689. 10.1021/jacs.1c04984. 34284578

[ref51] Zhao Y.; Zhao X.; Zang Y.; Di C.; Diao Y.; Mei J. Conjugation-Break Spacers in Semiconducting Polymers: Impact on Polymer Processability and Charge Transport Properties. Macromolecules 2015, 48 (7), 2048–2053. 10.1021/acs.macromol.5b00194.

[ref52] Zhao X.; Zhao Y.; Ge Q.; Butrouna K.; Diao Y.; Graham K. R.; Mei J. Complementary Semiconducting Polymer Blends: The Influence of Conjugation-Break Spacer Length in Matrix Polymers. Macromolecules 2016, 49 (7), 2601–2608. 10.1021/acs.macromol.6b00050.

[ref53] Sekitani T.; Nakajima H.; Maeda H.; Fukushima T.; Aida T.; Hata K.; Someya T. Stretchable Active-Matrix Organic Light-Emitting Diode Display Using Printable Elastic Conductors. Nat. Mater. 2009, 8 (6), 494–499. 10.1038/nmat2459. 19430465

[ref54] Shin M.; Oh J. Y.; Byun K.-E.; Lee Y.-J.; Kim B.; Baik H.-K.; Park J.-J.; Jeong U. Polythiophene Nanofibril Bundles Surface-Embedded in Elastomer: A Route to a Highly Stretchable Active Channel Layer. Adv. Mater. 2015, 27 (7), 1255–1261. 10.1002/adma.201404602. 25581228

[ref55] Liu D.; Ding Z.; Wu Y.; Liu S. F.; Han Y.; Zhao K. In Situ Study of Molecular Aggregation in Conjugated Polymer/Elastomer Blends toward Stretchable Electronics. Macromolecules 2022, 55 (1), 297–308. 10.1021/acs.macromol.1c01537.

[ref56] Xu J.; Wu H.-C.; Zhu C.; Ehrlich A.; Shaw L.; Nikolka M.; Wang S.; Molina-Lopez F.; Gu X.; Luo S.; Zhou D.; Kim Y.-H.; Wang G.-J. N.; Gu K.; Feig V. R.; Chen S.; Kim Y.; Katsumata T.; Zheng Y.-Q.; Yan H.; Chung J. W.; Lopez J.; Murmann B.; Bao Z. Multi-Scale Ordering in Highly Stretchable Polymer Semiconducting Films. Nat. Mater. 2019, 18 (6), 594–601. 10.1038/s41563-019-0340-5. 30988452

[ref57] Liu D.; Miao Q. Recent Progress in Interface Engineering of Organic Thin Film Transistors with Self-Assembled Monolayers. Mater. Chem. Front. 2018, 2 (1), 11–21. 10.1039/C7QM00279C.

[ref58] Zhang Z.; Wang W.; Jiang Y.; Wang Y.-X.; Wu Y.; Lai J.-C.; Niu S.; Xu C.; Shih C.-C.; Wang C.; Yan H.; Galuska L.; Prine N.; Wu H.-C.; Zhong D.; Chen G.; Matsuhisa N.; Zheng Y.; Yu Z.; Wang Y.; Dauskardt R.; Gu X.; Tok J. B.-H.; Bao Z. High-Brightness All-Polymer Stretchable LED with Charge-Trapping Dilution. Nature 2022, 603 (7902), 624–630. 10.1038/s41586-022-04400-1. 35322250

[ref59] Jiang Y.; Zhang Z.; Wang Y.-X.; Li D.; Coen C.-T.; Hwaun E.; Chen G.; Wu H.-C.; Zhong D.; Niu S.; Wang W.; Saberi A.; Lai J.-C.; Wu Y.; Wang Y.; Trotsyuk A. A.; Loh K. Y.; Shih C.-C.; Xu W.; Liang K.; Zhang K.; Bai Y.; Gurusankar G.; Hu W.; Jia W.; Cheng Z.; Dauskardt R. H.; Gurtner G. C.; Tok J. B.-H.; Deisseroth K.; Soltesz I.; Bao Z. Topological Supramolecular Network Enabled High-Conductivity, Stretchable Organic Bioelectronics. Science 2022, 375 (6587), 1411–1417. 10.1126/science.abj7564. 35324282

[ref60] Jeong M. W.; Ma J. H.; Shin J. S.; Kim J. S.; Ma G.; Nam T. U.; Gu X.; Kang S. J.; Oh J. Y. Intrinsically Stretchable Three Primary Light-Emitting Films Enabled by Elastomer Blend for Polymer Light-Emitting Diodes. Sci. Adv. 2023, 9 (25), eadh1504 10.1126/sciadv.adh1504. 37343088 PMC10284558

[ref61] Ha H.; Kim J.; Shim Y. J.; Irfan A.; Nimbalkar A.; Elumalai R.; Le T. N.; Kim H.; Suh M. C. Intrinsically Stretchable Emissive Layer for Green and Red Phosphorescent OLEDs: Small Molecules Blended with SEBS Elastomer. Adv. Mater. Technol. 2023, 8 (21), 2300924 10.1002/admt.202300924.

[ref62] Guo S.; Tong Y.; Wang X.; Zhang M.; Yu H.; Ren H.; Tang Q.; Lu G.; Liu Y. Brittle PCDTPT Based Elastic Hybrid Networks for Transparent Stretchable Skin-Like Electronics. Adv. Electron. Mater. 2023, 9 (9), 2200438 10.1002/aelm.202200438.

[ref63] Kim J. S.; Jeong M. W.; Nam T. U.; Vo N. T. P.; Jung K. H.; Lee T. I.; Oh J. Y. Intrinsically Stretchable Subthreshold Organic Transistors for Highly Sensitive Low-Power Skin-Like Active-Matrix Temperature Sensors. Adv. Funct. Mater. 2024, 34 (1), 2305252 10.1002/adfm.202305252.

[ref64] Peng Z.; Li S.; Zhou K.; Zhang Y.; Li M.; Li X.; Yang C.; Bian F.; Geng Y.; Ye L. Unveiling the Strain-induced Microstructural Evolution and Morphology-Stretchability Correlations of Intrinsically Stretchable Organic Photovoltaic Films. Adv. Energy Mater. 2024, 14 (18), 2304286 10.1002/aenm.202304286.

[ref65] Qin M.; Bian Y.; Wang C.; Sun J.; Shi W.; Liu K.; Zheng Y.; Zhang F.; Liu G.; Shao M.; Wen W.; Zhu Z.; Zhu M.; Zhao Z.; Wang H.; Liu Y.; Yuan G.; Guo Y. Intrinsically Stretchable Organic Photodiodes for Faint Near-Infrared Light Detection and Extendable Cryptographic Imaging. Adv. Funct. Mater. 2024, 34, 2403770 10.1002/adfm.202403770.

[ref66] Zhu C.; Wu H.-C.; Nyikayaramba G.; Bao Z.; Murmann B. Intrinsically Stretchable Temperature Sensor Based on Organic Thin-Film Transistors. IEEE Electron Device Lett. 2019, 40 (10), 1630–1633. 10.1109/LED.2019.2933838.

[ref67] Bian Y.; Liu K.; Ran Y.; Li Y.; Gao Y.; Zhao Z.; Shao M.; Liu Y.; Kuang J.; Zhu Z.; Qin M.; Pan Z.; Zhu M.; Wang C.; Chen H.; Li J.; Li X.; Liu Y.; Guo Y. Spatially Nanoconfined N-Type Polymer Semiconductors for Stretchable Ultrasensitive X-Ray Detection. Nat. Commun. 2022, 13 (1), 7163 10.1038/s41467-022-34968-1. 36418862 PMC9684452

[ref68] Zhang T.; Liu Y.; Zhang L.; Wang S.; Li J.; Zuo J.; Yu X.; Zhang Q.; Han Y. Constructing a Desired Nanofibril Network Morphology for Stretchable Polymer Films by Weakening the Intermolecular Interaction of a Conjugated Polymer in an Elastomer Matrix and Extending the Film-Forming Time. J. Mater. Chem. C 2023, 11 (6), 2302–2315. 10.1039/D2TC04896E.

[ref69] Lee J.; Nguyen T. H.; Oh E. S.; Lee S.; Choi J.; Kwon H. S.; Wang C.; Lee S.; Lee J.; Kim T.; Kim B. J. Establishing Co-Continuous Network of Conjugated Polymers and Elastomers for High-Performance Polymer Solar Cells with Extreme Stretchability. Adv. Energy Mater. 2024, 14, 2401191 10.1002/aenm.202401191.

[ref70] Nam T. U.; Vo N. T. P.; Jeong M. W.; Jung K. H.; Lee S. H.; Lee T. I.; Oh J. Y. Intrinsically Stretchable Floating Gate Memory Transistors for Data Storage of Electronic Skin Devices. ACS Nano 2024, 18 (22), 14558–14568. 10.1021/acsnano.4c02303. 38761154

[ref71] Park Y.; Fuentes-Hernandez C.; Kim K.; Chou W.-F.; Larrain F. A.; Graham S.; Pierron O. N.; Kippelen B. Skin-like Low-Noise Elastomeric Organic Photodiodes. Sci. Adv. 2021, 7 (51), eabj6565 10.1126/sciadv.abj6565. 34910518 PMC8673773

[ref72] Guan C.; Xiao C.; Liu X.; Hu Z.; Wang R.; Wang C.; Xie C.; Cai Z.; Li W. Non-Covalent Interactions between Polyvinyl Chloride and Conjugated Polymers Enable Excellent Mechanical Properties and High Stability in Organic Solar Cells. Angew. Chem., Int. Ed. 2023, 62 (44), e202312357 10.1002/anie.202312357. 37702544

[ref73] Liu J.; Wang J.; Zhang Z.; Molina-Lopez F.; Wang G.-J. N.; Schroeder B. C.; Yan X.; Zeng Y.; Zhao O.; Tran H.; Lei T.; Lu Y.; Wang Y.-X.; Tok J. B.-H.; Dauskardt R.; Chung J. W.; Yun Y.; Bao Z. Fully Stretchable Active-Matrix Organic Light-Emitting Electrochemical Cell Array. Nat. Commun. 2020, 11 (1), 3362 10.1038/s41467-020-17084-w. 32620794 PMC7335157

[ref74] Vo N. T. P.; Nam T. U.; Jeong M. W.; Kim J. S.; Jung K. H.; Lee Y.; Ma G.; Gu X.; Tok J. B.-H.; Lee T. I.; Bao Z.; Oh J. Y. Autonomous Self-Healing Supramolecular Polymer Transistors for Skin Electronics. Nat. Commun. 2024, 15 (1), 3433 10.1038/s41467-024-47718-2. 38653966 PMC11039670

[ref75] Jeon K.-H.; Park J.-W. Light-Emitting Polymer Blended with Elastomers for Stretchable Polymer Light-Emitting Diodes. Macromolecules 2022, 55 (18), 8311–8320. 10.1021/acs.macromol.2c01095.

[ref76] Wang Y.; Chen K.; Prine N.; Rondeau-Gagné S.; Chiu Y.; Gu X. Stretchable and Self-Healable Semiconductive Composites Based on Hydrogen Bonding Cross-linked Elastomeric Matrix. Adv. Funct. Mater. 2023, 33 (42), 2303031 10.1002/adfm.202303031.

[ref77] Zhang T.; Zhang L.; Wang S.; Li J.; Zuo J.; Chen R.; Yu X.; Peng J.; Zhang Q.; Han Y. Enhancing the Enrichment of Conjugated Polymer at Surfaces of Its Elastomer Blend Film via Increasing Molecular Weight to Induce Desired Nanofibril Network for Highly Stretchable Polymer Film. Polymer 2023, 278, 125986 10.1016/j.polymer.2023.125986.

[ref78] Ding Y.; Zhu Y.; Wang H.; Wang Y.; Gu X.; Wang X.; Qiu L. Improving Electrical and Mechanical Properties of Blend Films via Optimizing Solution-Processable Techniques and Controlling the Semiconductor Molecular Weight. Macromolecules 2022, 55 (19), 8577–8589. 10.1021/acs.macromol.2c00765.

[ref79] Peña-Alcántara A.; Nikzad S.; Michalek L.; Prine N.; Wang Y.; Gong H.; Ponte E.; Schneider S.; Wu Y.; Root S. E.; He M.; Tok J. B. -H.; Gu X.; Bao Z. Effect of Molecular Weight on the Morphology of a Polymer Semiconductor-Thermoplastic Elastomer Blend. Adv. Electron. Mater. 2023, 9 (9), 2201055 10.1002/aelm.202201055.

[ref80] Nikzad S.; Wu H.-C.; Kim J.; Mahoney C. M.; Matthews J. R.; Niu W.; Li Y.; Wang H.; Chen W.-C.; Toney M. F.; He M.; Bao Z. Inducing Molecular Aggregation of Polymer Semiconductors in a Secondary Insulating Polymer Matrix to Enhance Charge Transport. Chem. Mater. 2020, 32 (2), 897–905. 10.1021/acs.chemmater.9b05228.

[ref81] Huang W.; Liu X.; Ding Z.; Wang Z.; Xu C.; Li R.; Wang S.; Wu Y.; Qin R.; Han Y.; Geng Y.; Liu S. F.; Han Y.; Zhao K. Aligned Conjugated Polymer Nanofiber Networks in an Elastomer Matrix for High-Performance Printed Stretchable Electronics. Nano Lett. 2024, 24 (1), 441–449. 10.1021/acs.nanolett.3c04248. 38109494

[ref82] Chang Y.; Huang Y.-H.; Lin P.-S.; Hong S.-H.; Tung S.-H.; Liu C.-L. Enhanced Electrical Conductivity and Mechanical Properties of Stretchable Thermoelectric Generators Formed by Doped Semiconducting Polymer/Elastomer Blends. ACS Appl. Mater. Interfaces 2024, 16 (3), 3764–3777. 10.1021/acsami.3c15651. 38226590

[ref83] Kang H.; Lee Y.; Lee G. H.; Chung J. W.; Kwon Y.; Kim J.; Kuzumoto Y.; Gam S.; Kang S.; Jung J. Y.; Choi A.; Yun Y. Strain-Tolerant, High-Detectivity, and Intrinsically Stretchable All-Polymer Photodiodes. Adv. Funct. Mater. 2023, 33 (13), 2212219 10.1002/adfm.202212219.

[ref84] Shi H.; Liu C.; Jiang Q.; Xu J. Effective Approaches to Improve the Electrical Conductivity of PEDOT:PSS: A Review. Adv. Electron. Mater. 2015, 1 (4), 1500017 10.1002/aelm.201500017.

[ref85] Kayser L. V.; Lipomi D. J. Stretchable Conductive Polymers and Composites Based on PEDOT and PEDOT:PSS. Adv. Mater. 2019, 31 (10), 1806133 10.1002/adma.201806133. PMC6401235 30600559

[ref86] Bai Y.; Li W.; Tie Y.; Kou Y.; Wang Y.; Hu W. A Stretchable Polymer Conductor Through the Mutual Plasticization Effect. Adv. Mater. 2023, 35 (38), 2303245 10.1002/adma.202303245. 37318149

[ref87] Kim Y.; Yoo S.; Kim J.-H. Water-Based Highly Stretchable PEDOT:PSS/Nonionic WPU Transparent Electrode. Polymers 2022, 14 (5), 949 10.3390/polym14050949. 35267772 PMC8912668

[ref88] Li P.; Du D.; Guo L.; Guo Y.; Ouyang J. Stretchable and Conductive Polymer Films for High-Performance Electromagnetic Interference Shielding. J. Mater. Chem. C 2016, 4 (27), 6525–6532. 10.1039/C6TC01619G.

[ref89] Wang Y.; Zhu C.; Pfattner R.; Yan H.; Jin L.; Chen S.; Molina-Lopez F.; Lissel F.; Liu J.; Rabiah N. I.; Chen Z.; Chung J. W.; Linder C.; Toney M. F.; Murmann B.; Bao Z. A Highly Stretchable, Transparent, and Conductive Polymer. Sci. Adv. 2017, 3 (3), e1602076 10.1126/sciadv.1602076. 28345040 PMC5345924

[ref90] Tran H.; Feig V. R.; Liu K.; Wu H.-C.; Chen R.; Xu J.; Deisseroth K.; Bao Z. Stretchable and Fully Degradable Semiconductors for Transient Electronics. ACS Cent. Sci. 2019, 5 (11), 1884–1891. 10.1021/acscentsci.9b00850. 31807690 PMC6891860

[ref91] Oh J. Y.; Son D.; Katsumata T.; Lee Y.; Kim Y.; Lopez J.; Wu H.-C.; Kang J.; Park J.; Gu X.; Mun J.; Wang N. G.-J.; Yin Y.; Cai W.; Yun Y.; Tok J. B.-H.; Bao Z. Stretchable Self-Healable Semiconducting Polymer Film for Active-Matrix Strain-Sensing Array. Sci. Adv. 2019, 5 (11), eaav3097 10.1126/sciadv.aav3097. 31723597 PMC6839939

[ref92] Zheng Y.; Yu Z.; Zhang S.; Kong X.; Michaels W.; Wang W.; Chen G.; Liu D.; Lai J.-C.; Prine N.; Zhang W.; Nikzad S.; Cooper C. B.; Zhong D.; Mun J.; Zhang Z.; Kang J.; Tok J. B.-H.; McCulloch I.; Qin J.; Gu X.; Bao Z. A Molecular Design Approach towards Elastic and Multifunctional Polymer Electronics. Nat. Commun. 2021, 12 (1), 5701 10.1038/s41467-021-25719-9. 34588448 PMC8481247

[ref93] Zheng Y.; Michalek L.; Liu Q.; Wu Y.; Kim H.; Sayavong P.; Yu W.; Zhong D.; Zhao C.; Yu Z.; Chiong J. A.; Gong H.; Ji X.; Liu D.; Zhang S.; Prine N.; Zhang Z.; Wang W.; Tok J. B.-H.; Gu X.; Cui Y.; Kang J.; Bao Z. Environmentally Stable and Stretchable Polymer Electronics Enabled by Surface-Tethered Nanostructured Molecular-Level Protection. Nat. Nanotechnol. 2023, 18 (10), 1175–1184. 10.1038/s41565-023-01418-y. 37322142

[ref94] Tien H.; Li X.; Liu C.; Li Y.; He M.; Lee W. Photo-Patternable Stretchable Semi-Interpenetrating Polymer Semiconductor Network Using Thiol-Ene Chemistry for Field-Effect Transistors. Adv. Funct. Mater. 2023, 33 (15), 2211108 10.1002/adfm.202211108.

[ref95] Zheng Y.-Q.; Liu Y.; Zhong D.; Nikzad S.; Liu S.; Yu Z.; Liu D.; Wu H.-C.; Zhu C.; Li J.; Tran H.; Tok J. B.-H.; Bao Z. Monolithic Optical Microlithography of High-Density Elastic Circuits. Science 2021, 373 (6550), 88–94. 10.1126/science.abh3551. 34210882

[ref96] Li N.; Li Y.; Cheng Z.; Liu Y.; Dai Y.; Kang S.; Li S.; Shan N.; Wai S.; Ziaja A.; Wang Y.; Strzalka J.; Liu W.; Zhang C.; Gu X.; Hubbell J. A.; Tian B.; Wang S. Bioadhesive Polymer Semiconductors and Transistors for Intimate Biointerfaces. Science 2023, 381 (6658), 686–693. 10.1126/science.adg8758. 37561870 PMC10768720

[ref97] Wang S.; Urban M. W. Self-Healing Polymers. Nat. Rev. Mater. 2020, 5 (8), 562–583. 10.1038/s41578-020-0202-4.

[ref98] Perera M. M.; Ayres N. Dynamic Covalent Bonds in Self-Healing, Shape Memory, and Controllable Stiffness Hydrogels. Polym. Chem. 2020, 11 (8), 1410–1423. 10.1039/C9PY01694E.

[ref99] Wojtecki R. J.; Meador M. A.; Rowan S. J. Using the Dynamic Bond to Access Macroscopically Responsive Structurally Dynamic Polymers. Nat. Mater. 2011, 10 (1), 14–27. 10.1038/nmat2891. 21157495

[ref100] Li X.; Gong J. P. Role of Dynamic Bonds on Fatigue Threshold of Tough Hydrogels. Proc. Natl. Acad. Sci. U. S. A. 2022, 119 (20), e2200678119 10.1073/pnas.2200678119. 35549555 PMC9171766

[ref101] Cooper C. B.; Root S. E.; Michalek L.; Wu S.; Lai J.-C.; Khatib M.; Oyakhire S. T.; Zhao R.; Qin J.; Bao Z. Autonomous Alignment and Healing in Multilayer Soft Electronics Using Immiscible Dynamic Polymers. Science 2023, 380 (6648), 935–941. 10.1126/science.adh0619. 37262169

[ref102] Li C.-H.; Wang C.; Keplinger C.; Zuo J.-L.; Jin L.; Sun Y.; Zheng P.; Cao Y.; Lissel F.; Linder C.; You X.-Z.; Bao Z. A Highly Stretchable Autonomous Self-Healing Elastomer. Nat. Chem. 2016, 8 (6), 618–624. 10.1038/nchem.2492. 27219708

[ref103] Döhler D.; Kang J.; Cooper C. B.; Tok J. B.-H.; Rupp H.; Binder W. H.; Bao Z. Tuning the Self-Healing Response of Poly(Dimethylsiloxane)-Based Elastomers. ACS Appl. Polym. Mater. 2020, 2 (9), 4127–4139. 10.1021/acsapm.0c00755.

[ref104] Kang J.; Son D.; Wang G. N.; Liu Y.; Lopez J.; Kim Y.; Oh J. Y.; Katsumata T.; Mun J.; Lee Y.; Jin L.; Tok J. B. -H.; Bao Z. Tough and Water-Insensitive Self-Healing Elastomer for Robust Electronic Skin. Adv. Mater. 2018, 30 (13), 1706846 10.1002/adma.201706846. 29424026

[ref105] Rao Y.-L.; Chortos A.; Pfattner R.; Lissel F.; Chiu Y.-C.; Feig V.; Xu J.; Kurosawa T.; Gu X.; Wang C.; He M.; Chung J. W.; Bao Z. Stretchable Self-Healing Polymeric Dielectrics Cross-Linked Through Metal-Ligand Coordination. J. Am. Chem. Soc. 2016, 138 (18), 6020–6027. 10.1021/jacs.6b02428. 27099162

